# The Bacteria of Sewer Air

**Published:** 1909-10-02

**Authors:** 


### MEDICINE.

### THE BACTERIA OF SEWER AIR.

That nasty smells may produce disease has long been a popular belief: that nasty smells cannot by themselves cause illness is the view of most medical men. Many pathogenic organisms, particularly those of the coli group, produce gases which have nasty smells; the latter are danger signals, and no one would deny that it is wise to respect them. Nevertheless there has for a long time been a prevalent opinion in the profession that sewer gases do not contain bacteria, and that therefore nasty smells are not so dangerous as the laity believe. This view, however, is being rapidly disproved by workers such as Major Horrocks, of the Royal Army Medical Corps, and Dr. Andrewes for the Local Government Board. Some of the conclusions arrived at by the latter are of considerable practical importance. He uses special methods, which include timed exposures to the air to be tested of plates containing McConkey's medium of agar, bile salts, lactose and neutral red, in which bacillus coli colonies can readily be picked out by the development of red colour in them. It has been clearly proved that, far from being relatively innocuous, the air of drains and of sewers contains numbers of characteristic sewage bacteria under many ordinary circumstances. The streptococci of sewer air correspond in their characters with those of sewage rather than with those present in fresh air. Bacilli of the colon group, almost absent from fresh air, can be demonstrated in ordinary drain air with no great difficulty even when the drains are in perfectly good working order. It was shown by Major Horrocks and confirmed by Dr. Andrewes that bacillus prodigiosus, which is absent from ordinary sewage, and which may therefore be added to it as a test organism, can be recovered from the air of drains at a considerable distance from the point at which it was added. There is therefore nothing impossible in the view that typhoid bacilli may similarly be present in sewer air, and that typhoid fever may therefore be caught directly from " a bad smell."

It might be asked how the bacteria become dissociated from the fluid sewage so as to be present in the air. It would seem that there are at least three different ways in which this result might come about. These are: by the bursting of bubbles in stagnant or slowly flowing sewage; by splashing; and by the alternate moistening and drying of the walls of sewers and soil-pipes where the flow of sewage is intermittent. After a whole series of elaborate experiments, the conclusion arrived at is that the first and third of these are far less important than the second, namely, splashing as the fluid falls, or as the current in one pipe joins that in another. This is referred to as " droplet contamination," quite slight splashings leading to contamination of the adjacent air with droplets that are of minute size, probably less than 1/50,000 c.c. Even in the absence of demonstrable air currents, the influence of this droplet contamination extends for at least 14 feet in a horizontal direction and for at least 12 feet in a vertical direction, and this from a focus of but slight splashing or gurgling. In the presence of air currents the distances to which contaminating organisms might be carried would naturally be greater. It is a bad thing, therefore, to have drains that are either gurgly or draughty.

The other main factor besides splashing that determines the number of polluting organisms that even the best drain air contains is the actual amount of sewage matter that is passing through the pipes at a given time. This was confirmed by Dr. Andrewes in the cases of his own house and of St. Bartholomew's Hospital, in each of which instances the sanitary arrangements were as good as could be. The fact is of considerable importance, and it is most graphically expressed in Dr. Andrewes' own words: " I will deal first with the figures obtained in experiment vii, because the problem is reduced to its simplest terms in the drainage of a small house with only a few occupants. In this experiment, carried out on a Sunday, when domestic avocations are reduced to a minimum, I determined the hourly distribution of sewage bacteria in the air of the main inspection chamber from 6 A.M. to 11 P.M. From 6 to 7 A.M. the drains were absolutely quiescent, and the plate exposed showed no red colonies. At 7 A.M., or soon after, I had a bath, and the rest of the household began to arise, several baths being taken between 8 and 9 A.M. A few red colonies now appeared on the plates; between 7 and 8 A.M. there were five colonies, and between 8 and 9 A.M. nine colonies. Breakfast was at 9 A.M., and immediately afterwards the water-closets were in use. The plate exposed between 9 and 10 A.M. yielded 808 colonies, the number falling to 34 in the plate exposed from 10 to 11 A.M. The washing up incidental to breakfast was now over, and practically no colonies appeared on the plates till the time of the midday meal, when six was the maximum recorded for any single hour (1 to 2 P.M.). During the afternoon counts of four and five red colonies per hour were obtained, but after 4 o'clock the plates exposed were practically negative, in spite of an evening meal at 7 P.M. with its concomitant washing up, until the hours from 10 to 11 P.M., when five colonies appeared, explained by the fact that a bath was taken by one member of the household at that time.

" There is thus seen one enormous rise in the content of drain air in sewage bacteria, corresponding precisely to the hour at which the various members of the household had their bowels opened. The washing up after breakfast and the emptying of slops may have been greater than at later periods of the day, but cannot account for the inordinate rise at that particular hour. The experiment points very conclusively to the fæcal content of the sewage as the determining factor in the distribution of the colon bacillus and its allies in drain air."

The experiment was entirely confirmed by others. The possibility of drain air—even the air of drains that are not bad ones—spreading diseases is very clearly proved.

